# Establishing Concurrent Validity of the Role Checklist Version 2 with the OCAIRS in Measurement of Participation: A Pilot Study

**DOI:** 10.1155/2017/6493472

**Published:** 2017-01-22

**Authors:** Patricia J. Scott, Danielle Cacich, Morgan Fulk, Karen Michel, Katie Whiffen

**Affiliations:** ^1^Indiana University, Indianapolis, IN, USA; ^2^Franciscan Health, Indianapolis, IN, USA; ^3^NCA Therapy, Indiana University, Bloomington, IN, USA; ^4^Autism Treatment Center, San Antonio, TX, USA; ^5^Duke University Health System, Durham, NC, USA

## Abstract

Persons experiencing problems with adaptation following disease, disability, or overwhelming life circumstances are often referred by their physicians to occupational therapists. Given time constraints, therapists may skip administration of a client-centered participation focused assessment and instead use an impairment or limitation focused assessment. This approach assumes that skill remediation will naturally lead to return of participation in valued occupational roles because most participation measures take 30 minutes or longer. In response to the need for an efficient measure of desired role participation, this study establishes concurrent validity of the 10–15-minute Role Checklist Version 2 (RCV2: QP) with the 50 minute Occupational Circumstances Assessment And Rating Scale (OCAIRS) in measuring occupational participation in individuals recovering from surgery following liver transplantation. 20 subjects (mean age of 55 and a mean time-since-transplant of 5.2 months) completed both instruments. The hypothesis was supported (*r* = .63), showing concurrent validity between the OCAIRS and the RCV2: QP. This provides therapists with an efficient, client-centered measure of occupational participation for a client-centered treatment plan. Using the RCV2: QP in place of the OCAIRS provides a more efficient assessment tool for occupational therapists to set treatment goals and monitor client progress over time.

## 1. Introduction

Since its founding nearly 100 years ago, occupational therapy holds that following disease or disability return to productive and meaningful engagement in valued roles is the basis of the profession. Over time, role participation builds peoples' occupational identities and competence through ongoing adaptations to life changes. Identity and competence are challenged as life events unfold; they influence the state in which we find ourselves at any point in our lives [1]. Occupations are the things that we need to do and want to do every day. These include performance of basic everyday tasks such as dressing or eating, as well as our participation in more complex tasks such as working a job or investing in the stock market. Most individuals do not think much about the importance of participation in occupations until they experience an injury or disability that suddenly influences their ability to do so.

Persons often experience failures of the adaptation to life changes due to disease, disability, or overwhelming life circumstances. When this happens, their occupational identity and competence are threatened or compromised. These people frequently are referred to occupational therapy. Unfortunately, even though studies suggest that patients make more progress when they are given higher-level cognitive, motor, or task-specific interventions [2, 3], therapists often fail to assess role participation focusing on impairments or functional limitations [2–5].

Over time, the profession has developed several assessment tools to measure participation. Many of these assessment tools are administered during the initial evaluation; however, given time constraints, they compete with treatment time. This problem does not minimize the importance of measuring participation, but rather emphasizes the need to do so. Practitioners need a valid, reliable, and time efficient tool to measure occupational role participation in order to tailor therapy according to each patient's needs, as well as to measure effectiveness of interventions through monitoring resumption of desired roles. 

*The Model of Human Occupation (MOHO)*. The Model of Human Occupation (MOHO), one of the most commonly used theoretical models in occupational therapy worldwide, explains how individuals are motivated to engage in occupations, how occupations become habituated and performed in the context of individuals' environments, and how the performance of skills and processes are required to perform an occupation [1]. MOHO supports the premise that occupational role performance culminates in occupational participation as roles represent the intersection of one's identity and societal status. When people introduce themselves, it is often through an association with a role: a mother, student, carpenter, or lawyer. Each of these roles requires performance of a set of activities. For example, one may fulfill the role of a home maintainer, which includes the tasks of meal preparation, doing laundry, vacuuming, and money management. Role participation is predicated on occupational performance. Thus, tracking the return of roles from initial evaluation, throughout intervention, and at discharge is an important outcome measure in occupational therapy [6].

Based on the theoretical background of MOHO, a variety of assessments have been developed to measure participation in roles, including the 10–15 minute Role Checklist Version 2: (RCV2: QP) [6] and the more comprehensive 50 minute Occupational Circumstances Assessment Interview and Rating Scale Version 4 (OCAIRS V4) [7]. To test concurrent validity for a time efficient measure of occupational participation, both assessments were administered to sample patients who had undergone liver transplantation and were experiencing an extreme loss of role participation during end-stage liver disease [8].

Liver transplant recipients have nearly a 90% one-year survival rate [9]. While this survival rate is high, liver transplant patients experience lower rates of participation in valued roles and activities [8, 10, 11]. Scott [8] investigated the relationship between role participation and quality of life in 161 individuals on an average of five years after transplant. There was a significant decline in role participation for all subjects; the results revealed a strong correlation between the number of currently performed roles and quality of life. Therefore, if an individual has low role participation, it is likely he/she will also experience a lower quality of life.

*MOHO and Liver Transplant Patients*. MOHO guides therapists to understand how participation is influenced by personal factors, including volition, habituation, and performance capacity, as well as a significant external factor, the environment. An injury or illness such as receiving a liver transplant can influence these factors.

Volition is one's motivation to act and can be broken down into personal causation, values, and interests [1]. After a liver transplant, an individual's volition may be hindered by fatigue, causing decreased motivation to participate in various occupations. Personal causation refers to how competent and effective one feels [1]. For example, some liver transplant recipients may doubt their performance ability in various occupations after experiencing a major medical incident. The feeling of low self-efficacy may contribute to decreased participation in occupations.

Habituation refers to the patterns and routines people develop with everyday activities. A person's habits are drastically affected throughout all stages of the liver transplant process, from sickness before the transplant to returning home after transplantation. An individual who worked before liver transplant but does not return to work following transplantation may experience a change in sleep patterns due to a change in routine. The order and manner in which individuals get ready for the day may change, and a new routine may emerge due to medical appointments.

Perhaps the most direct impact a liver transplant has on an individual is on performance capacity. Performance capacity of an individual's performance capabilities is based upon his/her physical and mental capacity [1]. After the significant recovery period that occurs after liver transplantation, liver transplant recipients' bodies may seem foreign to them. Guided by MOHO, occupational therapists can enable liver transplant recipients to reclaim their bodies and identify new ways of performing their occupational roles [1].

In addition to individual factors, the environment also influences occupational participation. An example of environmental influence is social support. In some situations, an individual may discontinue a previously enjoyed hobby, such as traveling, for fear he/she would not have the stamina to do so. As stamina improves, adding additional supports of a spouse or friend to accompany the transplant recipient while traveling would enable the patient to resume the occupational role of a traveler. Thus, the environment can interact with a person's inner characteristics to determine one's capacity for role participation [1].

Because it breaks down overall participation into volition, habituation, and performance capacity, MOHO is an ideal framework for studying occupational role participation in liver transplant recipients. Each component of MOHO is influenced during the liver transplant process. On a broader scale, occupational therapists need to be able to determine how a client is participating in all occupations. Rooted in the MOHO framework, assessment tools including the RCV2: QP and the OCAIRS V4 were developed to measure occupational participation [1].

*Summary*. The Model of Human Occupation (MOHO) explains how individuals are motivated to engage in occupations and how these occupations become habituated and performed in their usual environment through roles [1]. MOHO defines occupational participation as “levels of doing” and provides assessments to measure these levels as well other salient concepts. The goal of this study is to determine whether the RCV2: QP and the OCAIRS V4 measure the construct of occupational participation in the same way. If concurrent validity is established between these two assessments, occupational therapists can confidently utilize the RCV2: QP in place of a longer assessment to more efficiently measure occupational participation. The findings of the current study can be used to create goals within an intervention plan aimed at improving occupational participation. The primary research question was as follows: is there a sufficiently strong relationship between scores on the RCV2: QP and the OCAIRS V4 to establish concurrent validity for measuring occupational participation as defined by MOHO? It is hypothesized that the OCAIRS V4 would have a strong correlation with the RCV2: QP, meaning that these two instruments similarly measure the concept of occupational participation.

## 2. Methods

In this pilot study, both the RCV2: QP and the OCAIRS V4 were given to 20 liver transplant recipients within the first year after transplant.

### 2.1. Participants

Participants were liver transplant patients recruited at Indiana University Health Hospital. Eligibility criteria included having received a liver transplant within the past 12 months, speaking English, being 18 years or older, and having the ability to demonstrate sound cognitive ability by completing the EncephalApp_Stroop test. Exclusion criteria included receiving more than one liver transplant. Potential participants were contacted by phone. After the nature of the study had been described and if verbal consent was received, potential participants were scheduled for 50 minutes before or after their medical appointments at Indiana University Health Hospital to complete the study. This study was approved by the Indiana University IRB # 1403154786.

### 2.2. Procedures

Following written informed consent, participants completed the RCV2: QP and the OCAIRS V4. Two researchers for each participant conducted interviews.

#### 2.2.1. Instrumentation

Standardized instruments included the RCV2: QP, the OCAIRS V4, and the EncephalApp. The EncephalApp_Stroop is a cognitive screening tool and the RCV2: QP and the OCAIRS V4 are measures of occupational participation.

*(1) EncephalApp_Stroop*. The EncephalApp_Stroop is a measure of psychomotor speed and cognitive flexibility [12]. This 5–7-minute test is administered on an iPad. This standardized assessment can be used as a cognitive screen for the presence or absence of dementia or Hepatic Encephalopathy (HE), both of which have the potential to prevent an individual from providing reliable data in the RCV2: QP: QP, OCAIRS V4, and interview. 

*(2) Role Checklist Version 2: Quality of Performance*. The RCV2: QP is a short screening tool that measures past, present, and future participation in valued roles. This assessment allows practitioners to gather patients' occupational participation and role performance in order to write client-centered, occupation-based goals. The reliability and content validity of the RCV2: QP are established [13] and this tool was found to be a valid measure of the construct of participation [14]. It is necessary to establish the concurrent validity of this instrument with another instrument measuring occupational participation within the MOHO framework.

The RCV2: QP is a short written or electronic assessment that measures role participation. It is based on Oakley et al.'s 1986 Role Checklist [6] that consists of two parts. Part one lists ten roles (student, worker, volunteer, caregiver, home maintainer, friend, family member, religious participant, and hobbyist, participation in organizations). The participant selects whether they have occupied each role at least once a week in the past, present, or future; all applicable options can be selected. It serves to capture one's perceived role incumbency or one's belief that he or she occupies a role and whether that role is part of one's self-identity. Part one is closely related to the MOHO construct of habituation. In part two, the client selects whether they find each of the ten roles “not at all valuable,” “somewhat valuable,” or “very valuable,” regardless of whether they have ever occupied that role. Part two aims to gather data on role value or the importance an individual attaches to a role. Part two ties into the construct of volition from the MOHO, describing how one's values impact their occupational behavior and satisfaction.

Scott [15] created Part 3 and Part 4, therefore creating RCV2: QP, which measures quality of performance, or a self-rating of satisfaction, to include the final construct from MOHO, performance capacity. The RCV2: QP contextualizes performance from the patient's perspective by having them indicate their level of satisfaction or dissatisfaction with involvement in a role. Additionally, the RCV2: QP contextualizes how accepting an individual is of decreased participation in future roles. Occupational therapists are interested in helping individuals achieve their highest possible quality of occupational participation for increased quality of life, making Parts 3 and 4 especially relevant in providing occupational therapy services. Using the RCV2: QP, occupational therapists can capture their patients' return to participation over time.

The RCV2: QP has clinical utility in that it informs occupational therapy practitioners of a client's priorities and satisfaction related to roles which can then be translated into therapy goals [16]. Perhaps more importantly, it can show a scarcity of roles, conflicts in role demands, or role imbalances, which may result in maladaptive occupational behavior. The RCV2: QP can serve to measure broad occupational participation at initial evaluation and can further be used in goal-setting and treatment planning. In order to assist with goal-setting and treatment planning, the RCV2: QP breaks down occupational roles into their performance components, which can be addressed as short-term goals during intervention sessions.

At reevaluation or discharge, the RCV2: QP reflects how occupational participation has improved. The RCV2: QP can be administered electronically using a tablet or computer. An email can be sent with a link to the assessment, so it can be completed by a client outside of therapy time, making it an even more time efficient tool.

The primary change from the original Role Checklist to the RCV2: QP includes the addition of self-reported rating of the quality of performance as compared to prior levels. Scott et al. (2014) have established the reliability and validity of the electronic administration of the RCV2: QP. For part one, kappa values for individual roles ranged from 0.53 to 0.95. Cronbach's alpha values of percent agreement between paper and electronic administrations on parts two (role value) and three (quality of participation) were significant with almost perfect agreement of 0.88 to 1.0. Thus, this assessment is both reliable and valid as administered in either written or electronic format to gain information about role incumbency, role value, and role performance. 

*(3) Occupational Circumstances Assessment Interview and Rating Scale V4 (OCAIRS V4)*. The Occupational Circumstances Assessment Interview and Rating Scale Version 4 (OCAIRS V4) is another instrument used to measure occupation participation. It consists of a semistructured interview, rating scale, and summary form. There are three versions of the OCAIRS V4: Mental Health, Forensic Mental Health, and Physical Disability. For the purposes of this study, the Physical Disability version will be utilized.

The OCAIRS V4 specifies sample questions for each section of the interview, and the interviewer is encouraged to ask additional questions to gain clarity of information and to ensure all necessary information is provided [17]. Additionally, the person conducting the interview makes it as conversational as possible to fit with the client and environment. The purpose of the interview is to collect information in the 12 following areas: roles, habits, personal causation, values, interests, skills, short-term goals, long-term goals, interpretation of past experiences, physical environment, social environment, and readiness for change. The individual administering the interview scores the client's responses by selecting one of four scores in each of the 12 categories. Each category is scored as either facilitates, allows, inhibits, or restricts participation in occupation. Each of the four score categories also has two to three descriptors to aid the interviewer in the selection of the most appropriate score. Upon completion of the interview, the therapist uses the four-point rating scale to develop an evaluation of the client's occupational profile. The OCAIRS V4 requires approximately 50 minutes to be completed, including both the interview and the rating [7].

At the time the OCAIRS was created in 1994, psychometric testing found interrater reliability of the fourteen items in the original OCAIRS using an intraclass correlation coefficient for items which ranged from 0.318 to 0.812. The two fair ICC values are for component score: system trajectory (*r* = 0.32) and feedback (*r* = 0.40). The two moderate correlations are for Global Assessment: historical (*r* = 0.57) and Physical Environment (*r* = 0.59). The remaining ten items range from *r* = 0.63 to *r* = 0.95. Ten of fourteen items had substantial or almost perfect interrater reliability [18]. The system trajectory score is designed to obtain information about past adaptive behavior and the historical score is a reflection of past adaptive capacity.

Concurrent validity was established between the OCAIRS and the Assessment of Occupational Functioning, another measure designed to measure occupational participation, and based on MOHO, with a high correlation of *r* = 0.86 [18]. Raw scores from the OCAIRS V4 were entered into the MOHO Clearinghouse database for electronic scoring.

### 2.3. Scoring

Scoring of parts 1 and 2 of the RCV2: QP mirrored previous findings by Scott [19]. The RCV2: QP yields nominal and ordinal data. See Table 1 for the coding system. Roles were coded “yes,” = 1, while roles coded “no” = 0. Ordinal ranking was used for the value section of the RCV2: QP, utilizing the following codes: “very valuable” = 3, “somewhat valuable” = 2, and “not at all valuable” = 1. Each presently performed role was multiplied by its value to establish a “weighted role.”

To score Part 3, a secondary calculation was performed using the weighted value for each role as currently occupied multiplied by a value (1, 2, or 3) and multiplied again by the quality of participation (worse = −1, same = 0, and better = 1). The choice of −1, 0, and 1 for ratings was to establish “0” as the desired norm and look at the movement of performance from this point.

The RCV2: QP can serve to measure broad occupational participation at initial evaluation and can further be used in goal-setting and treatment planning. In order to assist with goal-setting and treatment planning, the RCV2: QP breaks down occupational roles into their performance components, which can be addressed as short-term goals during intervention sessions.

The scoring for the OCAIRS follows the manual [7]. For each of the 12 scoring categories of OCAIRS V4, values are assigned as follows: facilitates = 4, allows = 3, inhibits = 2, and restricts = 1. The average of all categories is compiled for each subject to obtain an overall mean score.

### 2.4. Data Analysis

The nonparametric Spearman rank order correlation was used to examine the relationship between the OCAIRS V4 and the RCV2: QP. Data was analyzed using SPSS 22.

## 3. Results

Twenty participants (*N* = 20), including four females (*n* = 4) and 16 males (*n* = 16), completed the study (mean age = 55.1 years, SD = 10.7). The sample is similar in age to the population of US adult liver transplant recipients in 2014 (UNOS, 2014). Time after transplant ranged from two to 12 months with a mean of 5.2 months (SD = 3.23). For gender, our proportion of males was higher (Table 2).

### 3.1. RCV2: QP and OCAIRS V4

The RCV2: QP scores ranged from −1.2 to 3.0 with a mean of 0.56 and a standard deviation of 1.34. The OCAIRS V4 scores ranged from 2.08 to 4.0 with a mean of 3.43 and a standard deviation of 0.57. A Spearman rank order correlation between the OCAIRS V4 and the RCV2: QP found *r*(18) = .63 and *p* < .01 between composite OCAIRS V4 and RCV2: QP scores.

In order to investigate whether there was an effect on days-since-transplant on scores on either the RCV2: QP or the OCAIRS V2, a Spearman rank order correlation was calculated for each. There was no significant correlation between days-since-transplant on either the RCV2: QP (*r*(18) = −.21, *p* = .39) or the OCAIRS V4 (*r*(18) = −.13, *p* = .59).

## 4. Discussion

This pilot study aimed to establish concurrent validity of the RCV2: QP and the OCAIRS V4 by determining if they measure occupational participation as defined by MOHO, in post-liver-transplant patients. The hypothesis that the two instruments measure the construct of participation was supported. The RCV2: QP is a multipurpose utility measure in that it is time efficient, has flexible administration, and can be used throughout the occupational therapy process in this small sample of liver transplant patients.

With the knowledge that the RCV2: QP produces similar results as the more time intensive OCAIRS V4, occupational therapy practitioners will now be able to quickly determine how patients are progressing in their pursuit to occupy desired roles. In addition to being more time efficient, the RCV2: QP is also convenient as it can be administered electronically, through the mail, or over the phone [13]. The RCV2: QP measures broad occupational participation at the initial evaluation and can further be used in goal-setting and treatment planning by breaking down occupational roles into performance components. At reevaluation or discharge, the RCV2: QP can be administered again to see how role participation has improved.

### 4.1. Limitations and Future Directions

A concern worth addressing is the two fair and moderate ICC values from the OCAIRS. These values are for component score: physical environment (*r* = 0.59) and feedback (*r* = 0.40), Global Assessment: system trajectory (*r* = 0.32), and historical (*r* = 0.57). The RCV2: QP is not intended to measure the environment or assess the impact of past events on the individual. When used clinically to set treatment goals, this is matter for discussion. The RCv3 instead is intended to screen patients for satisfaction with current roles; the OCAIRS is a more comprehensive instrument. One exciting finding is the ICC for the Component scale for internalized roles—internalized roles being a key construct for the RCV2: QP which is *r* = 0.95.

The small sample of participants and specific population limits generalizability of these findings. Replication with other populations of individuals recovering from other disabling conditions such as stroke, cancer, or spinal cord injury who experience disruption in occupational participation can further demonstrate the validity of the Role Checklist revisions. Additionally, given the Role Checklist has no scoring system and researchers have applied various methods to score, the RCV2: QP will benefit from a scoring system.

### 4.2. Conclusion and Implications for Practice

The MOHO “levels of doing” include occupational skills, occupational performance, and occupational participation. Occupational skills assist with the completion of the occupations needed for role performance and thus enable role participation. Although we recognize the differences between the general RCV2: QP and the more detailed OCAIRS V4, both instruments measure occupational participation.

Occupational therapists can use the RCV2: QP to reveal client reports of both desired role participation as well as decreased participation. Both of these situations are indications for the need for occupational therapy services. Occupational therapists can intervene by addressing the inner characteristics, such as are volition, habituation, and performance capacity of the patient with an overall goal of increasing role participation.

Ideally, the occupational therapy process begins with identification of a client's desire to return to participation in valued roles. However, due to the burden of assessment, therapists often gravitate towards a limitation focused approach in both their evaluations and their interventions, focusing on skill deficits instead of participation. Therefore, there is a need of an efficient and client-centered measure of occupational participation that a therapist can utilize in a realistic amount of time. The RCV2: QP fulfills this need; it measures participation at the beginning of therapy to identify participation goals and can continue to be used to assess the treatment effectiveness to accomplish patients' goals. This research exemplifies that there is a strong correlation between the OCAIRS V4 and the RCV2: QP. By having established concurrent validity as well as other previously established psychometric properties, practitioners can confidently administer the RCV2: QP as a time efficient yet accurate measure of occupational participation.

## Figures and Tables

**Table 1:** Scoring of the RCV2: QP as used in this study.

RCV3 parts	Responses	Scoring
Part 1: are you presently performing “*X*” role	Yes = 1 No = 0	For each role the respondent says yes (currently performing), the “1” is multiplied for the value assigned to that same role Example: currently performing work = 1 (yes), value = 3 (very valuable) following Scott et al. [13]

Part 2: is this role valuable	Very valuable = 3 Somewhat valuable = 2 Not at all valuable = 1	

Part 3: please rate the quality of your participation	Worse = −1 Same = 0, Better = 1	The score from Parts 1 and 2 is multiplied by the rating of quality of performance applied to the above example: currently performing work = 1 (yes), *X* value = 3 (very valuable) = 6; on Part 3 respondents rated quality as same, so we have (6 × 0) = 0

**Table 2:** Demographic characteristics of the study sample and the United Network for Organ Sharing (UNOS) sample of all adults who received liver transplants in 2014 Showing no significant differences in age or in gender.

	UOS population		Sample population	
	(*n* = 6199)		(*n* = 20)	
	*n*	Proportion	*n*	Proportion
Gender^1^				
Female	2065	33.31	4	20
Male	4134	66.69	16	80
Age group^2^				
18–34	392	6.32	1	5
35–49	927	14.95	3	15
50–64	3826	61.72	13	65
65+	1054	17.00	3	55

^1^Fisher exact value is *p* = .24.

^2^The 4 × 2 Chi-square goodness of fit test is *p* = .09.
